# Acute bilateral blindness in a young Covid-19 patient with rhino-orbito-cerebral mucormycosis

**DOI:** 10.1186/s12348-021-00272-0

**Published:** 2021-10-18

**Authors:** Ines Malek, Jihene Sayadi, Rim Lahiani, Miriam Boumediene, Memia Ben Salah, Myriam Jrad, Moncef Khairallah, Leila Nacef

**Affiliations:** 1 A Department Hedi Raies Institute of Ophthalmology, Tunis El-Manar University, Tunis, Tunisie; 2 Department of Oto-rhino-laryngology, Charles Nicoles Hospital, Tunis El-Manar University, Tunis, Tunisia; 3 Department of Radiology, Charles Nicoles Hospital, Tunis El-Manar University, Tunis, Tunisia; 4 Department of Ophthalmology, Fattouma Bourguiba University Hospital, Faculty of Medicine, University of Monastir, Monastir, Tunisia

## Case report

A diabetic 20-year-old male patient, not previously vaccinated against SARS-CoV-2, was referred to our emergency department for rapid bilateral visual loss with left periorbital pain, proptosis, palpebral edema and swelling.

He had been admitted one week before in a primary Covid-19 center for COVID-19 respiratory distress syndrome and treated with corticosteroids.

**Fig. 1:**
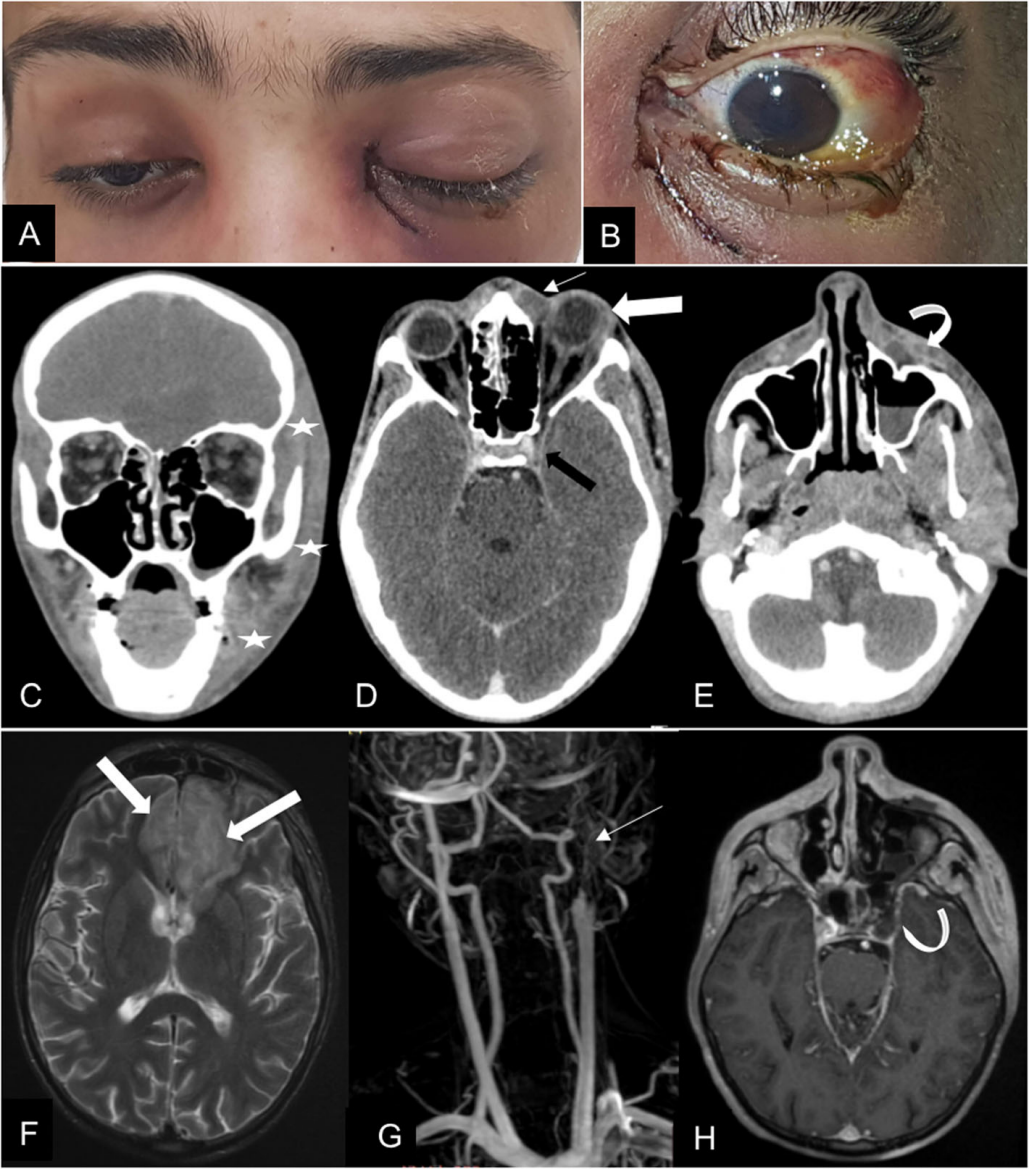
A 20-year old COVID-19-affected patient presented with rhino-orbitocerebral mucormycosis. (**A**) Complete blepharoptosis and inflammatory signs are seen on the left side. There is a mild proptosis on the right side. (**B**) Note the conjunctival injection, the chemosis and the corneal oedema. (**C**) Coronal and (**D**, **E**) Axial contrast-enhanced brain CT scan show thickening with infiltration of left hemiface fat planes (white stars); left pre-septal collection (arrow) with exophthalmos and lengthening of the antero-posterior axis of the eyeball (large arrow). This collection reaches the cellulo-fatty tissues next to the left maxillary sinus (curved arrow), the latter is the site of a partial liquid filling. The left cavernous sinus shows signs of thrombosis (black arrow). (**F**) axial T2 weighted image MRI showing T2 hypersignals (large arrow) in areas of the frontal lobes confirming endocranial extension. (**G**) MR angiography shows left carotid artery thrombosis (arrow). (**H**) 3D T1 weighted sequence with contrast shows left cavernous sinus thrombosis (curved arrow)

**Fig. 2:**
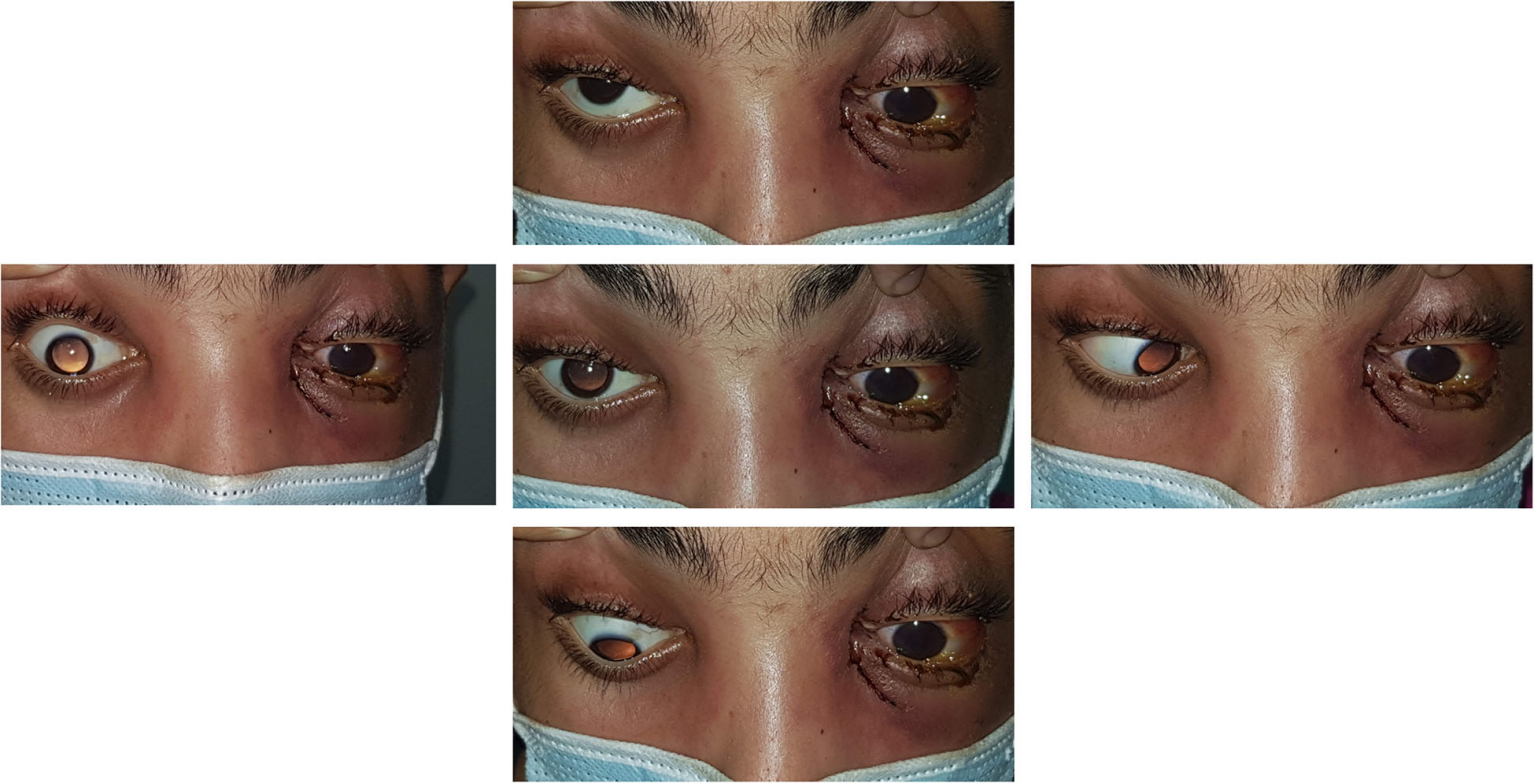
Clinical photographies showing complete left ophthalmoplegia and limited right eye abduction

**Fig. 3:**
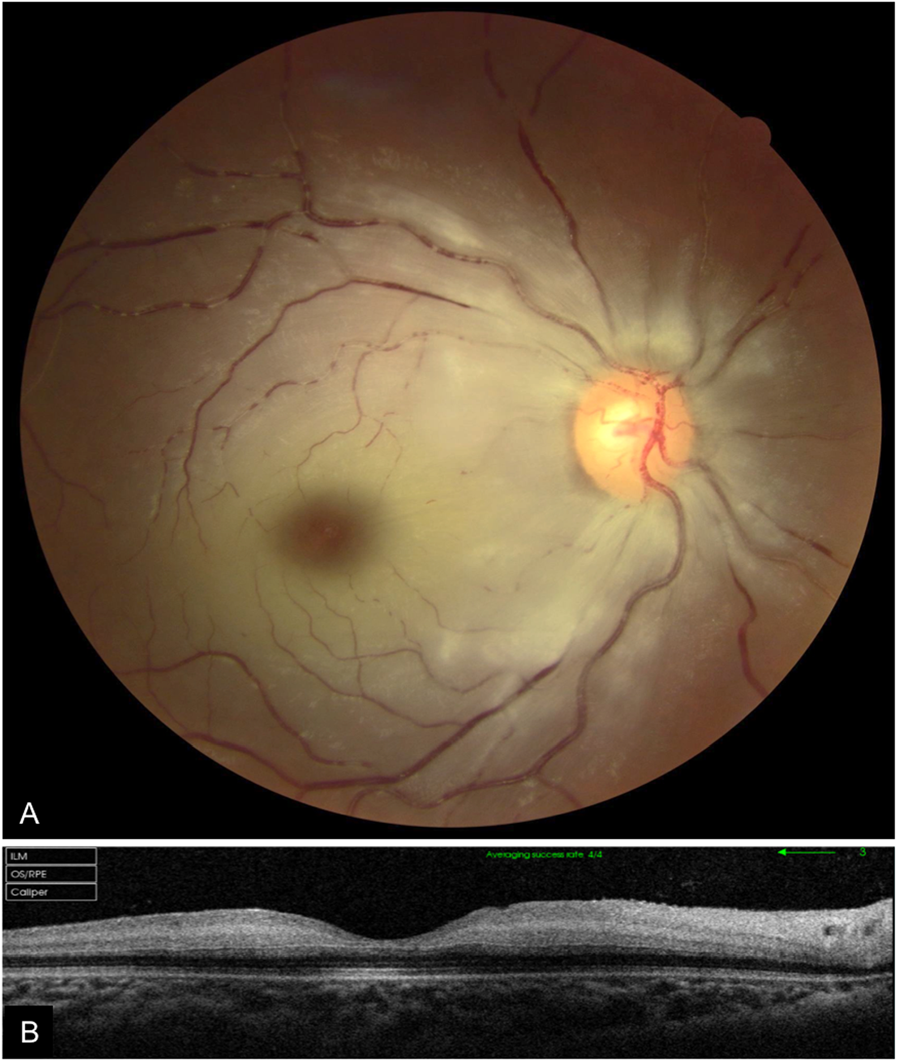
(**A**) Fundus photography of the right eye showing retinal whitening with a cherry red spot and segmental blood flow consistent with central retinal artery occlusion. (**B**) Swept source OCT scan showing hyperreflectivity of inner retinal layers

Upon ophthalmic examination, both eyes had a fixed dilated pupil with no light perception. The left eye showed features of orbital cellulitis (Fig. 1) with complete ophthalmoplegia (Fig. 2). There was a mild proptosis and limited abduction in the right eye (Fig. 2), and fundus examination showed retinal whitening with a cherry red spot and segmental blood flow consistent with central retinal artery occlusion (CRAO) (Fig. 3). Swept source OCT showed in the right eye hyperreflectivity of inner retinal layers corresponding to ischemic edema (Fig. 3).

Brain CT complemented with cerebral MRI disclosed endocranial extension of the disease and thrombosis of both left cavernous sinus and left internal carotid artery (Fig. 1).

The patient underwent emergency endoscopic sinus examination and removal of a blackish necrotic tissues from paranasal sinuses. Histopathological examination confirmed the diagnosis of mucormycosis.

The patient was started on intra-venous liposomal amphotericin B and clavulanic-acid-amoxicillin. Dexamethasone was discontinued. Debridement of the involved sinuses and adjacent structures was attempted together with diluted amphotericin B irrigation. Orbital exenteration was discussed but it was not retained due to poor prognosis and lack of the patient’s consent.

**Fig. 4:**
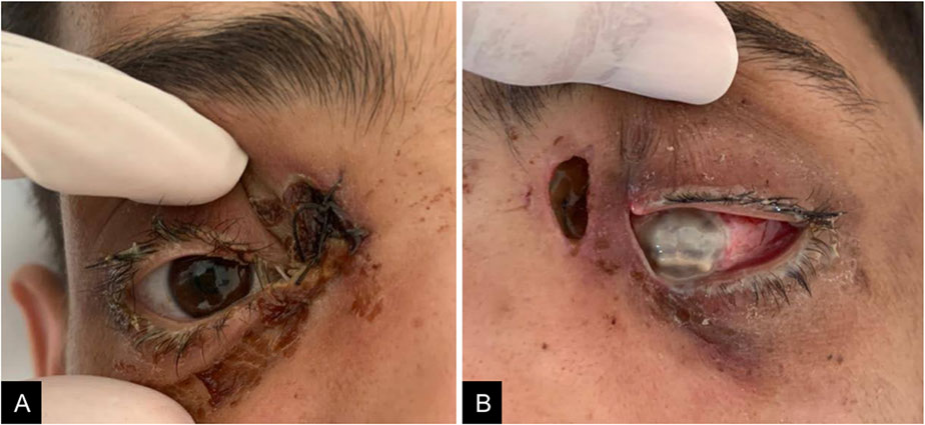
Clinical photographies 12 days after initial presentation showing bilateral extensive eyelid necrosis with purulent melting of the left eyeball

Over the following days, the patient’s ocular and general conditions worsened due to bilateral extensive eyelid and facial necrosis with purulent melting of the left eyeball and central nervous system involvement (Fig. 4). He passed away at day 30.

## Discussion

With the onset of COVID-19 pandemic, clinicians have seen a sudden surge of cases of mucormycosis. Although all ocular structures may be involved in patients with COVID-19 infection, orbital mucormycosis seems to be the most aggressive and so far fatal complication [1–3].

To the best of our knowledge very few cases of severe bilateral blindness in Covid-19 patient have been reported [2–6]. They were related either to orbital location of mucormycosis [2, 3] or to neurological complications including bilateral optic neuritis [4] and occipital ischemic stroke [5, 6].

The presentation of our patient suggests a left cavernous sinus and internal carotid thrombosis together with a right CRAO. From the ophthalmic and other orbital arteries, fungal infection advanced into the left cavernous sinus, ipsilateral carotid artery and right central retinal artery. This resulted in acute onset of bilateral vision loss and left ophthalmoplegia.

Successful treatment of ROCM highly depends on the early diagnosis of the infection, the control of the underlying predisposing factors and aggressive surgery [7].

Hence, Ophthalmologists should have a high index of suspicion for mucormycosis development in diabetic patients with COVID-19 illness, treated with corticosteroids. Vaccination against SARS-COv2 seems to be the best treatment to prevent COVID-19 and related blinding and fatal diseases.

## Data Availability

For data, please refer to corresponding author.

## Abbreviations

ROCM: Rhino-Orbito-Cerebral Mucormycosis
CRAO: Central Retinal Artery Occlusion
